# Ovarian hyperstimulation syndrome in a spontaneous singleton pregnancy

**DOI:** 10.1590/S1679-45082016RC3429

**Published:** 2016

**Authors:** Fábio Roberto Cabar

**Affiliations:** 1 Elo Clínica de Saúde, São Paulo, SP, Brazil.

**Keywords:** Ovarian hyperstimulation syndrome/etiology, Pregnancy trimester, first, Pregnancy complications, Ovarian cysts, Case reports

## Abstract

The ovarian hyperstimulation syndrome is the combination of increased ovarian volume, due to the presence of multiple cysts and vascular hyperpermeability, with subsequent hypovolemia and hemoconcentration. We report a case of spontaneous syndrome in a singleton pregnancy. This was a spontaneous pregnancy with 12 weeks of gestational age. The pregnancy was uneventful until 11 weeks of gestational age. After that, the pregnant woman complained of progressive abdominal distention associated with abdominal discomfort. She did not report other symptoms. In the first trimester, a routine ultrasonography showed enlarged ovaries, multiples cysts and ascites. Upon admission, the patient was hemodynamically stable, her serum β-hCG was 24,487mIU/mL, thyroid-stimulating hormone was 2.2µUI/mL and free T4 was 1.8ng/dL. All results were within normal parameters. However, levels of estradiol were high (10,562pg/mL). During hospitalization, she received albumin, furosemide and prophylactic dose of enoxaparin. The patient was discharged on the sixth hospital day.

## RESUMO

A síndrome de hiperestimulação ovariana é a combinação do aumento dos ovários, devido à presença de múltiplos cistos e de hiperpermeabilidade vascular, com subsequente hipovolemia e hemoconcentração. Relata-se um caso de síndrome espontânea em uma gestação única. Trata-se de gravidez espontânea com 12 semanas de idade gestacional. A gravidez ocorreu sem intercorrências até 11 semanas de idade gestacional. Após, a gestante passou a se queixar de distensão abdominal progressiva, associada com desconforto abdominal. A paciente não relatava outros sintomas. A ultrassonografia de rotina no primeiro trimestre mostrou ovários aumentados com múltiplos cistos e ascite. No momento da internação, a paciente apresentava-se hemodinamicamente estável, com β-hCG sérico de 24.487mUI/mL, hormônio estimulante da tireoide de 2,2µUI/m e T4 livre de 1,8ng/dL, ou seja, valores dentro dos parâmetros normais. Porém, os níveis de estradiol estavam elevados (10.562pg/mL). Durante a internação, a paciente recebeu albumina, furosemida e enoxaparina profilática. A alta hospitalar ocorreu no sexto dia de internação.

## INTRODUCTION

The ovarian hyperstimulation syndrome (OHSS) is the combination of increased ovarian volume, due to the presence of multiple cysts and vascular hyperpermeability, that results in the outflow of fluid from the intravascular space, with subsequent hypovolemia and hemoconcentration.^(^ ^1^ ^)^

In most cases, the OHSS is an iatrogenic complication of ovulation induction. Severe OHSS has incidence of 1 to 2% in superovulation cycles, and it remains one of the most important complications related with gonadotropin use in assisted reproductive technologies. The severe type is associated with morbidity, but rarely with mortality.^(^ ^2^ ^)^ Infrequently, it may be associated with spontaneous ovulatory cycles. This syndrome is generally described in multiple, hypothyroidism and molar pregnancies.^(^ ^3^ ^)^

The symptoms of spontaneous OHSS develop later than in iatrogenic OHSS: the syndrome occurs between 3 to 5 weeks of amenorrhea in iatrogenic cycle, and between 8 and 12 weeks of amenorrhea in spontaneous OHSS cases.^(^ ^4^ ^)^

The OHSS can be classified in mild, moderate and severe based on the gravity of signs, symptoms, laboratory tests and ultrasound findings. Severe OHSS is characterized by enlarged ovary (largest diameter greater than 12cm), presence of numerous ovarian cysts, ascites and, sometimes, pleural and/or pericardial effusion. Some cases may present electrolyte imbalance (hyponatremia and hyperkalemia), hypovolemic shock, renal failure, thromboembolism, and death.^(^ ^5^ ^)^

We report a case of spontaneous OHSS in a singleton pregnancy.

## CASE REPORT

This was a 25-year-old nulliparous woman, with a spontaneous pregnancy, 12 weeks of gestational age according to the last menstrual period. The pregnancy was uneventful until 11 weeks of gestational age. After that, the woman started to complain of progressive abdominal distention associated with abdominal discomfort, but no other symptoms were reported.

The first trimester routine ultrasonography showed enlarged ovaries, multiples cysts and ascites.

There was no reference to illness. Menstrual cycles were irregular without dysmenorrhea, acne or hirsutism. There were no records of gynecological diseases.

Upon admission, the patient was hemodynamically stable. Her pelvic examination showed uterus size compatible with gestational age, and large and mobile bilateral adnexal masses.

Laboratorial exams showed hemoglobin level of 13.1g/dL, hematocrit of 39.5%, hypoalbuminemia of 2.3g/dL, and hypoproteinaemia of 5.2g/dL; coagulation tests, hepatic and renal function had no changes (urea of 29mg/dL and creatinine of 0.8mg/dL). Serum β-hCG was 24,487mIU/mL, thyroid-stimulating hormone (TSH) was 2.2μUI/ml and free T4 was 1.8ng/dL. All results were within normal parameters. However, levels of estradiol were higher (10,562pg/mL).

Transvaginal sonography revealed a single live fetus at 12 weeks and normal amniotic fluid. The patient’s ovaries were enlarged (right ovary was 13.9cm and the left ovary was 12.8cm measured from their major axis) with multiple cysts, and moderate amount of pelvic fluid. The ultrasound screening for chromosomal abnormalities was negative.

During hospitalization, she was under clinical and sonographic surveillance and received intravascular human albumin for 3 days, furosemide (40mg/day) and prophylactic dose of enoxaparin. She remained clinically stable, and recovery was seen in serum exams and sonographic parameters. The patient was discharged on the sixth day of hospitalization. At hospital discharge, the ultrasound revealed large ovaries, the right with 9.3cm and the left with 8.1cm measured from their the major axis. There were no signs of ascites.

The pregnancy continued without other complications. A male newborn (birth weight 3,125kg) was born at 38 weeks and 5 days of gestational age by vaginal delivery. No complications occurred after the delivery.

Ten days of postpartum, the patient was asymptomatic, and at the sixth week after delivery she started to use intramuscular medroxyprogesterone acetate depot for contraception. No alterations were seen at physical examination. Pelvic sonography showed regular and uniform size uterus, a thin and regular endometrium; her ovaries were normal.

## DISCUSSION

In general, spontaneous OHSS develops between 8 and 14 weeks of amenorrhoea, which differ from iatrogenic OHSS that usually start between 3-5 weeks of amenorrhoea. Spontaneous forms of OHSS are extremely rare and always seen during pregnancy. A number of cases have been seen during multiple pregnancies or hydatidiform moles, which are reported to be associated with abnormally high values of hCG. Other cases are associated with hypothyroidism suggesting that the high levels of TSH could stimulate the ovaries.

The pathophysiological mechanism of the OHSS is not fully explained. It seems to depend on the release of vasoactive substances secreted by the ovaries, causing mesothelial hyperpermeability with fluid leakage from the intravascular to the extravascular space, therefore, generating a massive third space. The crux constitutes an equilibrium between proangiogenic and antiangiogenic factors found in follicular fluid. The hCG, estradiol, prolactin, histamine and prostaglandins have all been implicated in OHSS. But today we understand better that vasoactive substances such as interleukins, tumor necrosis factor alpha (TNF-α), endothelin-1 and vascular endothelial growth factor (VEGF) released by the ovaries have been involved in increasing vascular permeability.^(^ ^3^ ^,^ ^6^ ^)^

The loss of fluid and protein into the abdominal cavity generates hypovolemia and hemoconcentration responsible for blood circulation disorders and renal function. The most severe complications are the result of hypercoagulability and decreased renal perfusion.

The syndrome intensity is related to the degree of ovarian follicular response. Estrogens produced by the developed follicles reach very high levels and can be used as markers of degree of hyperstimulation.

In most cases, the OHSS results from administration of exogenous gonadotropins for infertility treatments. However, in some circumstances, this syndrome occurs in the absence of such treatment. A number of authors suggest that this syndrome occurrence is more frequent in cases of polycystic ovary syndrome, hypothyroidism, twin pregnancy and molar pathology.^(^ ^3^ ^)^ In our case, the pregnant woman have none of these alterations.

Ovarian cancer should be part of the differential diagnosis in cases of rapid ovarian growth. However, bilateral involvement and ultrasonography images of simple cysts and homogeneous thin wall, even with standard physiologic vascularization, suggest a benign dysfunction.^(^ ^7^ ^)^ Measurement of serum CA-125 has little relevance because its value is usually high during pregnancy. To determine estradiol levels might be the key of the diagnosis. Estradiol levels greater than 6,000pg/mL, as well as the characteristics of the ultrasound ovarian masses were consistent with diagnosis of OHSS.^(^ ^8^ ^)^

The cause of spontaneous ovarian hyperstimulation appears to be a permittivity of ovarian follicle-stimulating hormone (FSH) receptor for hCG and/or TSH. Three mechanisms are described in the genesis of spontaneous OHSS. There have been reports of spontaneous OHSS associated with high levels of hCG in particular molar pregnancies and multiple births. This mechanism is related with the hyperactivation of the FSH receptor in granulosa cells of the ovary by hCG, with consequent ovarian hyperstimulation. Similarly, spontaneous elevated TSH by hyperactivation of FSH from the TSH receptor, as occurs in hypothyroidism, could cause the syndrome. The third mechanism is based on the mutation of the FSH receptor, with consequent decrease of its specificity and increased sensitivity to hCG and TSH.^(^ ^9^ ^,^ ^10^ ^)^

De Leener^(^ ^11^ ^)^ classified spontaneous OHSS syndrome into three types based on clinical presentation and FSH receptor mutation. Type I is attributed to the mutated FSH receptor and seems to cause recurrent spontaneous OHSS. Type II is secondary to high levels of hCG as in hydatiform mole and multiple gestation and it is the most common one. Type III is associated with hypothyroidism. Mutations in the follicle-stimulating hormone receptor (FSHR) might be activated, which lead to a predisposition to OHSS, or inactivated, resulting in sterility due to poor ovarian response to gonadotropins. Polymorphisms of FSHR have been studied and about 744 single nucleotide polymorphisms have been identified observed in the FSHR gene. Of these polymorphisms, only eight were found in the coding region, exons, and the remaining are intronic. Ovarian response dependent on the FSHR genotype. Other studies on the p.N680S polymorphism of the FSHR gene have reported the homozygous Ser/Ser variant to be less sensitive to endogenous or exogenous FSH in terms of oestradiol production. Polymorphism of the FSHR, Ser680Asn, in the FSHR gene, is a predictor of the severity of symptoms in patients who develop OHSS.^(^ ^12^ ^,^ ^13^ ^)^

Activating mutations of the FSHR gene that cause ovarian hyper-responsiveness for circulating FSH or even cross-responsiveness of FSHR to hormones with similar structure to FSH, such as hCG or TSH,^(^ ^14^ ^)^ and hCG/LH receptor gene mutation claimed increased response to normal hCG levels and, therefore, ovarian hyper responsiveness.^(^ ^11^ ^)^

In our case, the values of TSH and hCG were normal. The most likely mechanism is the mutation of the FSH receptor gene. This diagnosis is possible by genetic sequencing, because several mutations in the gene have been described. The presence of mutation has implications regarding the recurrence of the syndrome in future pregnancies.^(^ ^10^ ^)^ Despite ovarian volume and moderate ascites, the patient had few symptoms which allowed a conservative approach with human albumin infusion only.
